# Smoking, distress and COVID-19 in England: Cross-sectional population surveys from 2016 to 2020

**DOI:** 10.1016/j.pmedr.2021.101420

**Published:** 2021-05-29

**Authors:** Loren Kock, Jamie Brown, Lion Shahab, Graham Moore, Marie Horton, Leonie Brose

**Affiliations:** a Department of Behavioural Science and Health, University College London, WC1E 7HB, United Kingdom; b SPECTRUM Research Consortium, Edinburgh, United Kingdom; c DECIPHer, School of Social Sciences, Cardiff University, Cardiff, United Kingdom; d Population Health Analysis Team, Public Health England, United Kingdom; e Institute of Psychiatry, Psychology and Neuroscience, King’s College London, United Kingdom

**Keywords:** Smoking, Mental health, COVID-19

## Abstract

Changes in the prevalence of psychological distress among smokers during the COVID-19 pandemic in England may exacerbate existing health inequalities. This study examined the prevalence of psychological distress among smokers following the onset of the pandemic compared with previous years. Cross-sectional data came from a representative survey of smokers (18+) in England (n = 2,927) between April–July in 2016, 2017 and 2020. Logistic regressions estimated the associations between past-month distress across 2016/2017 and 2020, and age. Weighted proportions, chi-squared statistics and stratified logistic regression models were used to compare the distributions of moderate and severe distress, respectively, within socio-demographic and smoking characteristics in 2016/2017 and 2020. Between the combined April–July 2016 and 2017 sample and April–July 2020 the prevalence of moderate and severe distress among past-year smokers increased (2016/2017: moderate 20.66%, 19.02–22.43; severe 8.23%, 7.16–9.47; 2020: moderate 28.79%, 95%CI 26.11–31.60; OR = 2.08, 95%CI 1.34–3.25; severe 11.04%, 9.30–13.12; OR = 2.16, 1.13–4.07). While there was no overall evidence of an interaction between time period and age, young (16–24 years) and middle-age groups (45–54 years) may have experienced greater increases in moderate distress and older age groups (65+ years) increases in severe distress. There were increases of moderate distress among more disadvantaged social grades and both moderate and severe distress among women and those with low cigarette addiction. Between April–July 2016/2017 and April–July 2020 in England there were increases in both moderate and severe distress among smokers. The distribution of distress among smokers differed between 2016/2017 and 2020 and represents a widening of inequalities.

## Highlights

• Mental health of the general population has deteriorated since the onset of the COVID-19 pandemic.
• Smoking is strongly associated with poor mental health.
• Deterioration in mental health among smokers may exacerbate health inequalities.
• There were increases in psychological distress in smokers between 2016/17 and 2020.

## 1 Introduction

Between 2014 and 2015 the prevalence of adult smoking in England was estimated to be 16.4% (Public Health England, 2020). During the same period smoking prevalence was higher among those with anxiety or depression (28.0%), and serious mental illness (40.5%) (including but not limited to psychosis, bipolar disorder, eating disorders and severe depression) (Public Health England, 2020). Those with a mental health condition are more likely to be more dependent smokers and to have greater difficulty in remaining abstinent after quitting, despite greater desire to quit compared with the general population (Richardson et al., 2019). These differences in smoking may account for up to two thirds of the inequality in life expectancy between those living with and without a mental health condition (Tam et al., 2016). Psychological distress is defined as mental health problems that are severe enough to cause moderate to serious impairment in social or occupational functioning and require treatment (Pratt, 2001). This study aimed to examine the prevalence of psychological distress among smokers following the onset of the COVID-19 pandemic in England compared with previous years.

Psychological distress is more common among smokers and is negatively associated with quit success and abstinence (Lawrence et al., 2011, Streck et al., 2020). The relationship between smoking and psychological distress may be explained by common risk factors related to socio-economic position (Kim-Mozeleski et al., 2019), but research also suggests potential bidirectionality (Fluharty et al., 2017). Individuals may be motivated to smoke to alleviate symptoms of psychological distress, and there is evidence that smoking may itself directly increase the risk of it occurring (Boden et al., 2010, Skov-Ettrup et al., 2016).

Between 2016/2017 in England, 24.3% and 9.7% of past-year smokers indicated moderate and serious psychological distress in the past month, respectively (Brose et al., 2020). Also, those with an indication of a mental health problem were more dependent on cigarettes but more likely to be motivated and have recently attempted to quit.

The COVID-19 pandemic and resulting government ‘lockdown’ measures were associated with a deterioration of mental health in the UK compared with pre-COVID-19 trends (Pierce et al., 2020, Boland et al., 2017, Bishop, et al., 1998, Sweet, 2017). Research has suggested that following the March 2020 government restrictions, smokers were more likely to try and quit, and rates of smoking cessation were higher (Jackson et al., 2020). However, a deterioration in mental health among smokers may negatively impact quitting behaviour given that smokers with distress have been found to be less likely to quit and remain abstinent (Lawrence et al., 2011, Streck et al., 2020).

Our previous research has highlighted an age gradient in psychological distress among smokers, with younger groups reporting higher levels of distress compared with older age groups (Brose et al., 2020). However, considering the sharp positive age gradient in the risk of death from COVID-19 (Verity et al., 2020), deterioration in mental health during the pandemic may be more pronounced among older age groups.

An increase in the prevalence of psychological distress among smokers during the COVID-19 pandemic in England could potentially widen existing health inequalities. Monitoring levels of distress among smokers is important to highlight unmet need for mental health and smoking cessation support in general and also during current and future respiratory disease epidemics. Using Smoking Toolkit Study (STS) data the aims of this study were to i) examine the prevalence of psychological distress among past-year smokers during April–July 2020 compared with the same monthly time period (April–July) in 2016 and 2017 (the previous time distress was assessed in the STS) and ii) examine the distribution of psychological distress within sociodemographic and smoking characteristic categories of past-year smokers during April–July 2020 and April–July 2016 and 2017.

## 2 Methods

### 2.1 Study design

Data were drawn from the Smoking Toolkit Study (STS), a monthly repeated cross-sectional survey of a representative sample of adults in England (Fidler et al., 2011). The dataset for the primary analysis consisted of four months of STS data from April–July in each of the years 2016, 2017 and 2020. Respondents were age 18 years or older.

Each month, a form of random location in combination with quota sampling is used to select a new sample of approximately 1,700 adults aged 18 years and older. Further details on the design of the STS, including sampling and weighting technique can be found elsewhere (Fidler et al., 2011). Comparisons with other national surveys show that the STS recruits a representative sample of the population in England (Fidler et al., 2011). Data are usually collected monthly through face-to-face computer assisted interviews. However, due to the COVID-19 pandemic, from March 2020 data were collected via telephone only. Diagnostic analyses have suggested it is reasonable to compare data from before and after the lockdown, despite the change in data collection (Jackson et al., 2020).

Ethical approval for the STS is granted by the UCL Ethics Committee (ID 0498/001; ID: 2808/005). The Strengthening the Reporting of Observational Studies in Epidemiology (STROBE) reporting guideline were used in the design and reporting of this study (Knottnerus and Tugwell, 2008).

#### 2.1.1 Dependent variables, independent variables and covariates

##### 2.1.1.1 Dependent variable

The primary outcome of this study was the prevalence of psychological distress among past-year smokers. This was derived using the following measures.

Psychological distress

Psychological distress was measured using the K6 community screening measure of non-specific psychological distress in the past month (Kessler et al., 2002, Kessler et al., 2010). The K6 intends to identify people with a high likelihood of having a diagnosable mental illness and its associated functional effects using six questions. The K6 measures non-specific psychological distress and was developed to identify those with moderate to severe impairment in social, occupational functioning and to require treatment (Pratt, 2001). The measure has ‘substantial’ concordance with independent clinical ratings of serious mental illness (Kessler et al., 2010). The K6 has demonstrated utility to screen for severe psychological distress, but also for a moderate yet still clinically relevant level that warrants mental health intervention (Prochaska et al., 2012). Therefore, based on previous research scores >13 were categorised as severe psychological distress, scores between 5 and 12 as moderate and <5 as no/minimal psychological distress (See Appendix) (Prochaska et al., 2012).

##### 2.1.1.2 Independent variables

Smoking status

Smoking status was ascertained using responses to the following question:

“Which of the following best applies to you?”

Those who responded with “I smoke cigarettes (including hand rolled) every day” and “I smoke cigarettes (including hand rolled), but not every day” were categorised as current cigarette smokers.

Those who responded with “I smoke cigarettes (including hand rolled) every day”, “I smoke cigarettes (including hand rolled), but not every day” and “I have stopped smoking completely in the last year” were categorised as past-year smokers. Past-year smokers is an important categorisation because it includes current smokers and a minority of those who have recently quit, but who are very likely to relapse to current smoking within a year (Hughes et al., 2004).

Those indicating that they do not smoke cigarettes, but do smoke tobacco of some kind (e.g. Pipe, cigar or shisha) were excluded from the analysis (n = 138) because they do not include measures of dependence that are measured for cigarette smokers.

##### 2.1.1.3 Smoking and quitting behaviour

Cigarette addiction

Cigarette addiction was measured using the heaviness of smoking index (HSI) (Kozlowski et al., 1994). This HSI uses two questions from the Fagerström Test for Cigarette Dependence: time to first cigarette in the morning after waking and the number of cigarettes smoked per day. Those with a score >4 are considered to have high addiction, and those with <4 considered to have low/moderate addiction.

Motivation to stop smoking

Motivation to stop smoking was assessed using the Motivation To Stop Scale (Kotz et al., 2013), a single-item measure with seven response options representing increasing motivation to quit. Responses were collapsed into two variables reflecting high (6–7) vs. low or no motivation to stop smoking (1–5) (see Appendix) (Kotz et al., 2013).

Quit attempts

Quit attempts in the past month was measured among past year smokers using the question “How many serious attempts to stop smoking have you made in the last 12 months?”, and if one or more attempts were reported: “How long ago did your most recent serious quit attempt start?”.

We distinguished those who attempted to quit up to 1 month ago versus those who made no quit attempt or attempted to quit >1 month before the interview but were not successful.

Socio-demographic characteristics

The socio-demographic variables age, sex, occupation-based social grade, region of England, and the presence of children in the household were measured (see Appendix and Table 1).

Time period

**Table 1:** Characteristics of past-year smokers (weighted data) in 2016, 2017 and 2020.

**Characteristic**	**Total (n %)**	**Year**		
		2016 (%)	2017 (%)	2020 (%)
***Year***				
2016	1106 (34.44)	–	–	–
2017	1066 (33.20)	–	–	–
2020	1039 (32.36)	–	–	–

***Past-month psychological distress***				
None	2170 (67.58)	786 (71.05)	759 (71.19)	625 (60.17)
Moderate	748 (23.29)	234 (21.16)	215 (20.14)	299 (28.79)
Severe	293 (9.12)	86 (7.79)	92 (8.68)	115 (11.04)

***Age***				
18–24	556 (17.32)	189 (17.11)	187 (17.52)	180 (17.36)
25–34	822 (25.60)	265 (23.99)	238 (22.32)	318 (30.64)
35–44	579 (18.03)	208 (18.78)	198 (18.61)	173 (16.60)
45–54	545 (16.97)	190 (17.21)	211 (19.79)	143 (13.80)
55–64	376 (11.71)	129 (11.67)	125 (11.68)	122 (11.77)
65+	334 (10.40)	282 (11.23)	42 (10.07)	12 (9.83)

***Social grade***				
AB	481 (14.98)	164 (14.86)	146 (13.68)	171 (16.44)
C1	765 (23.82)	265 (23.97)	265 (24.89)	235 (22.60)
C2	780 (24.29)	288 (26.09)	254 (23.80)	238 (22.89)
D	681 (21.21)	214 (19.36)	225 (21.09)	242 (23.28)
E	504 (15.70)	174 (15.72)	176 (16.53)	154 (14.78)

***Sex***				
Women	1544 (48.08)	509 (46.00)	536 (49.74)	535 (48.56)

***Children in household***				
Yes	1143 (35.60)	407 (36.85)	371 (34.77)	365 (35.07)
No	2068 (64.40)	698 (63.15)	695 (65.23)	675 (64.93)

Unweighted n = 2,972. *Year = 4 month time period (April–July) of specified year. Social grade. AB = Higher managerial, administrative and professional; B = Intermediate managerial, administrative and professional; C1 = Supervisory, clerical and junior managerial, administrative and professional; C2 = Skilled manual workers; D = Semi-skilled and unskilled manual workers; E = State pensioners, casual and lowest grade workers, unemployed with state benefits only. Other = responses of “Men” or “In another way”. Data are from the Smoking Toolkit Study.

The variable for time period included four months of data (April–July) each from the years 2016, 2017 and 2020. In this study, data from 2016 and 2017 were collapsed together to form a new variable reflecting April–July 2016 and 2017. These time periods were chosen because questions related to mental health outcomes were not included in the surveys during 2018 and 2019 and were only re-added from April 2020. The years 2016 and 2017 were collapsed given the missing information from 2018 and 2019, which restricted our ability to do a complete trend analysis over the period and because the raw estimates for prevalence of moderate and severe psychological distress were similar in the years 2016 and 2017. The comparison of the same four month time period in 2020 and 2016/2017 sought to account for potential seasonality in mental health disorders (De Graaf et al., 2005, Magnusson, 2000).

#### 2.1.2 Sample selection

Overall, 19,960 (unweighted) adults aged 18 + were surveyed. Of these, 3,640 past-year (current and recent ex) smokers were asked the mental health questions. Those who did not complete the mental health questions or selected ‘I don’t know’ or ‘prefer not to say’ in response to any of them (n = 399), or had missing data on any of the other variables included in the present analysis were excluded. This left a final unweighted sample size for analysis of 2,972 past-year smokers of which 2,418 were current smokers.

#### 2.1.3 Statistical analysis

To address our first aim (to examine the prevalence of psychological distress among past-year smokers during April–July 2020 compared with the same monthly time period in 2016/2017), weighted proportions (95% CIs) were used to describe the prevalence of past-month moderate and severe psychological distress, respectively, during the period of April–July 2020, and April–July 2016 and 2017 among past-year smokers.

We constructed separate logistic regression models to assess prevalence of moderate and severe psychological distress (dependent variable), respectively, among smokers (past-year and current) between the two time periods (April–July 2020 vs April–July 2016 and 2017 as referent) and age (six categories with 16–24 as referent) and the interaction terms. The model including exclusively current smokers was included as a sensitivity analysis to assess whether the effect size for the odds of psychological distress in 2020 compared with 2016/2017 was affected by the recent ex-smokers in the past-year smoker sample.

All associations are reported as odds ratios (ORs) with 95% confidence intervals (adjusted for sex, social grade and region). The inclusion of the time period*age interaction allowed us to examine potentially differential changes in psychological distress over time at different levels of age, which is of interest given the strong age gradient in risk of death from COVID-19 (Verity et al., 2020).

To address our second aim (to examine the distribution of psychological distress within sociodemographic and smoking characteristic categories of past-year smokers during April–July 2020 and 2016/2017), we: i) calculated weighted proportions and chi-square statistics to compare the distribution of moderate and severe psychological distress, respectively, within socio-demographic (age, sex, social grade, whether there were children in the house) and smoking characteristics (cigarette addiction, quit attempts and motivation to stop smoking) of past-year smokers during April–July 2020 and April–July 2016 and 2017; and ii) constructed stratified logistic regression models to examine any differences within these socio-demographic and smoking characteristic sub-groups between April–July 2016 and 2017 (referent) and April–July 2020. All associations are reported as odds ratios (ORs) with 95% confidence intervals (adjusted for age, social grade, sex and region except where the covariate was the variable of interest).

Analysis was carried out in R version 3.6.0 in September 2020. A 2-sided P < .05 was considered statistically significant. The analysis plan was pre-registered online at https://osf.io/eh6sk/.

Sensitivity analysis

The same analyses reported for the primary analysis was conducted but comparing April–July 2020 with all months in 2016/2017.

Unregistered post-hoc analyses

We conducted further logistic regression models to explore differences in moderate and severe psychological distress among recent ex-smokers (quit within the past year) between the two time periods (April–July 2020 vs April–July 2016 and 2017 as referent) and age (see Appendix).

In 2016 and 2017 mental health data was only collected among current and recent ex-smokers. From April 2020 all respondents were asked questions about their mental health, allowing us to examine levels of psychological distress across all categories of smoking status (see Appendix).

### 2.2 Results

**Fig. 1:**
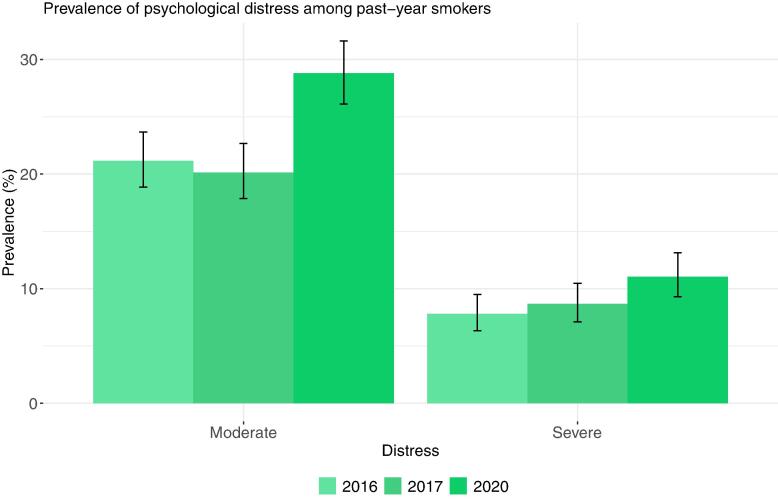
Prevalence of psychological distress among past-year smokers in 2016, 2017 and 2020 (weighted data).

A weighted total of 3,211 past-year smokers (mean (SD) age = 43.26 (17.11) years; 48.08% women) completed the STS survey between April–July in 2016 (n = 1,106), 2017 (n = 1,066) and 2020 (n = 1,039). Among the overall sample 748 (23.29%) reported moderate psychological distress, and 293 (9.12%) reported severe psychological distress. See Table 1 for an overview of the sample characteristics. Weighted prevalence statistics for moderate and severe psychological distress among past-year smokers in 2016, 2017 and 2020 are shown in Fig. 1. Data are from the University College London Smoking Toolkit Study in England.

#### 2.2.1 Psychological distress between 2016/2017 and 2020

Past-year smokers

Past-year smokers in 2020 had twice the odds of moderate and severe psychological distress, respectively, compared with 2016/2017 (Table 2). An age gradient was apparent, with older age groups less likely to report moderate or severe psychological distress, respectively, compared with those aged 16–24. There was no evidence of an interaction between time period and age.

**Table 2:** Associations between i) moderate (yes vs no) and ii) severe psychological distress (yes vs no) and time period of survey (April–July 2020 vs April–July 2016 and 2017) among past-year and current smokers in England.

	**Past-year smokers**				**Current smokers**			
	**Moderate distress**^a^	***P***	**Severe distress**^b^	***P***	**Moderate distress**^c^	***P***	**Severe distress**^d^	***P***
	(n = 2,706)		(n = 2,293)		(n = 2,455)		(n = 2,095)	
***Time period***								
2016 and 2017 ref	1 [Reference]		1 [Reference]		1 [Reference]		1 [Reference]	
2020	2.08 (1.34–3.25)	**0.001**	2.16 (1.13–4.07)	**0.02**	2.14 (1.31–3.50)	**0.002**	1.99 (0.95–4.01)	0.06

***Age***								
18–24	1 [Reference]		1 [Reference]		1 [Reference]		1 [Reference]	
25–34	0.67 (0.48–0.94)	**0.02**	0.60 (0.36–0.98)	**0.04**	0.63 (0.44–0.89)	**0.01**	0.57 (0.33–0.96)	**0.03**
35–44	0.67 (0.47–0.95)	**0.03**	0.68 (0.4–1.13)	0.14	0.67 (0.46–0.97)	**0.03**	0.75 (0.44–1.26)	0.28
45–54	0.46 (0.32–0.66)	**<0.001**	0.54 (0.31–0.90)	**0.02**	0.46 (0.32–0.68)	**<0.001**	0.5 (0.28–0.86)	**0.01**
55–64	0.46 (0.31–0.68)	**<0.001**	0.51 (0.29–0.88)	**0.02**	0.49 (0.32–0.72)	**<0.001**	0.45 (0.24–0.8)	**0.01**
65+	0.22 (0.14–0.34)	**<0.001**	0.12 (0.05–0.25)	**<0.001**	0.23 (0.14–0.36)	**<0.001**	0.12 (0.05–0.26)	**<0.001**

***Interaction terms***								
2020*25–34	0.79 (0.44–1.41)	0.42	0.91 (0.39–2.13)	0.82	0.78 (0.41–1.48)	0.45	1.03 (0.41–2.64)	0.95
2020*35–44	0.76 (0.39–1.45)	0.40	0.78 (0.31–1.99)	0.60	0.85 (0.42–1.73)	0.66	0.87 (0.32–2.4)	0.79
2020*45–54	0.95 (0.49–1.81)	0.87	0.55 (0.19–1.49)	0.25	0.93 (0.46–1.87)	0.83	0.62 (0.20–1.84)	0.39
2020*55–64	0.59 (0.3–1.17)	0.13	0.39 (0.13–1.11)	0.08	0.51 (0.24–1.06)	0.07	0.55 (0.18–1.68)	0.30
2020*65+	0.82 (0.38–1.75)	0.61	1.31 (0.38–4.65)	0.67	0.74 (0.32–1.66)	0.45	1.07 (0.27–4.20)	0.92

Ns are not weighted. All models are adjusted for age, sex and region.

a Sample includes past-year smokers with moderate (n = 679) and none/minimal (n = 2,027) psychological distress;

b Sample includes past-year smokers with severe (n = 266) and none/minimal (n = 2,027) psychological distress.

c Sample includes current smokers with moderate (n = 600) and none/minimal (n = 1,855) psychological distress;

d Sample includes current smokers with severe (n = 240) and none/minimal (n = 1,855) psychological distress. Models are adjusted for social grade, sex and region. Data are from the University College London Smoking Toolkit Study in England.

A sensitivity analysis using the entire two-year period of 2016 and 2017 as a comparator time period produced similar results to the main analysis (Appendix Table A1).

Current smokers

Similarly, current smokers in 2020 had twice the odds of moderate psychological distress compared with 2016/2017 (Table 2). An age gradient was also apparent among current smokers, with older age groups less likely to report moderate or severe psychological distress, respectively, compared with those aged 16–24. There was no evidence of an interaction between time period and age.

Recent ex-smokers

Among the much smaller group of recent ex-smokers (n = 277) there were no significant associations between moderate or severe psychological distress, respectively, in 2020 compared with 2016/2017 (Appendix Table A2). Based on observed increases in mental health problems pre and post COVID-19 among the general population in the UK (Daly et al., 2020), exploratory expected effect sizes (ORs) were set to 1.1, 1.5 and 1.9 respectively. The calculation of Bayes factors under all of these contexts indicated that the data were insensitive to detect these effects (Appendix Table A3).

Prevalence of psychological distress according to smoking status between April–July 2020

There were greater levels of both moderate and severe psychological distress among smokers, recent and >1 year ex-smokers compared with never smokers (Appendix Fig. A1 and Table A4).

#### 2.2.2 The distribution of psychological distress within sociodemographic and smoking characteristics of past-year smokers in 2016/2017 and 2020

Moderate psychological distress

The prevalence of moderate *psychological* distress was higher in 2020 compared with 2016/2017 among: those aged 16–24 and 45–54, women, those in more disadvantaged social grades, those with and without children in the house, and among those with low cigarette addiction (Table 3). No differences were apparent among those who had tried to quit within the past month or among current smokers with high motivation to quit.

Severe psychological distress

**Table 3:** The sociodemographic profile of i) moderate and ii) severe psychological distress in 2016/2017 and 2020 among past-year smokers.

	**Past year smokers experiencing distress**				**Past year smokers experiencing distress in 2020 compared with 2016/2017**	
	**2016/2017**	**2020**				
	**n (%)**	**n (%)**	**Χ**	***P***	**ORadj (95% CI) (2016/2017 ref vs 2020)**	***P***
**Moderate psychological distress**	429 (20.68)	250 (27.84)	–	–	–	–

***Age***						
16–24	112 (21.17)	56 (41.48)	**6.37**	**0.01**	**2.05 (1.32–3.18)**	**0.001**
25–34	96 (23.47)	68 (29.82)	**2.77**	**0.10**	**1.37 (0.93–2)**	**0.11**
35–44	76 (22.35)	37 (28.24)	1.49	0.22	1.44 (0.89–2.30)	0.13
45–54	62 (17.13)	39 (28.06)	**6.79**	**0.01**	**2.13 (1.3–3.48)**	**0.003**
55–64	51 (18.02)	30 (20.98)	0.36	0.55	1.17 (0.67–2.00)	0.58
65+	32 (10.81)	20 (16.39)	1.99	0.16	1.74 (0.9–3.29)	0.09

***Sex***						
Women	229 (22.79)	156 (32.98)	**16.83**	**<0.001**	**1.85 (1.44–2.38)**	**<0.001**
Other	200 (18.71)	94 (22.12)	2.02	0.15	1.27 (0.95–1.69)	0.10

***Social grade***						
AB	50 (17.92)	21 (14.29)	0.67	0.41	0.74 (0.4–1.32)	0.32
C1	111 (18.75)	89 (30.69)	**15.15**	**<0.001**	**2.31 (1.63–3.29)**	**<0.001**
C2	80 (17.86)	57 (28.64)	**8.97**	**0.003**	**1.86 (1.23–2.79)**	**0.003**
D	75 (19.33)	50 (34.72)	**13.00**	**<0.001**	**2.10 (1.35–3.26)**	**<0.001**
E	113 (30.79)	33 (27.97)	0.22	0.64	1.03 (0.63–1.65)	0.92

***Children in house***						
Yes	138 (20.18)	76 (26.76)	**4.68**	**0.03**	**1.57 (1.12–2.20)**	**0.01**
No	291 (20.94)	174 (28.34)	**12.69**	**<0.001**	**1.60 (1.27–2.02)**	**<0.001**

***HSI***						
Low (<4)	368 (20.18)	227 (27.92)	**18.87**	**<0.001**	**1.57 (1.29–1.91)**	**<0.001**
High (≥4)	61 (24.40)	23 (27.06)	0.12	0.73	1.24 (0.67–2.24)	0.48

***Quit attempt***						
In past month	31 (24.80)	7 (21.21)	0.04	0.84	0.98 (0.34–2.59)	0.98

MTSS*						
In ≤ 3 months	68 (24.29)	26 (21.85)	0.16	0.69	0.91 (0.53–1.51)	0.71

Severe psychological distress	174 (8.39)	92 (10.24)	–	–	–	–

***Age***						
16–24	47 (12.24)	20 (14.81)	0.38	0.54	1.55 (0.89–2.71)	0.12
25–34	35 (8.56)	28 (12.28)	1.88	0.17	1.22 (0.66–2.21)	0.52
35–44	32 (9.41)	17 (12.98)	0.94	0.33	1.73 (0.86–3.43)	0.12
45–54	29 (8.01)	10 (7.19)	0.01	0.90	1.23 (0.52–2.74)	0.62
55–64	24 (8.48)	9 (6.29)	0.37	0.55	0.85 (0.34–2.00)	0.72
65+	7 (2.36)	8 (6.56)	3.26	0.07	**3.31 (1.04–10.95)**	**0.04**

***Sex***						
Women	107 (10.65)	61 (12.90)	1.40	0.24	**1.52 (1.07–2.16)**	**0.02**
Other	67 (6.27)	31 (7.29)	0.37	0.54	1.24 (0.78–1.95)	0.36

***Social grade***						
AB	13 (4.66)	10 (6.80)	0.50	0.48	1.68 (0.67–4.13)	0.26
C1	37 (6.25)	24 (8.28)	0.95	0.33	1.52 (0.84–2.7)	0.16
C2	24 (5.36)	17 (8.54)	1.85	0.17	1.52 (0.76–2.97)	0.22
D	34 (8.76)	19 (13.19)	1.83	0.18	1.63 (0.86–3.06)	0.13
E	66 (17.98)	22 (18.64)	0.001	0.98	1.15 (0.65–2.00)	0.63

***Children in house***						
Yes	66 (9.65)	31 (10.92	0.23	0.63	1.39 (0.85–2.22)	0.18
No	108 (7.77)	61 (9.93)	2.31	0.13	1.38 (0.97–1.96)	0.07

***HSI***						
Low (<4)	137 (7.51)	78 (9.59)	2.99	0.08	**1.38 (1.02–1.86)**	**0.03**
High (≥4)	37 (14.80)	14 (16.47)	0.04	0.85	1.47 (0.69–3.03)	0.31

***Quit attempt***						
In past month	9 (7.20)	5 (15.15)	1.17	0.28	2.91 (0.73–11.2),	0.12

MTSS*						
In ≤ 3 months	22 (7.86)	9 (7.56)	<0.001	1.00	1.06 (0.44–2.37)	0.90

All logistic regression models assess levels of psychological distress in April–July 2020 compared with the referent of April–July in 2016 and 2017 within each sociodemographic characteristic (and are adjusted for age, social grade, sex and region except where the covariate was the variable of interest).

The chi-square analysis assesses the relationship between time period and distress within each group.

Ns are not weighted.

HSI = heaviness of smoking index. *MTSS = motivation to stop smoking. MTSS is measured among current cigarette smokers only.

Other = responses of “Men” or “In another way”.

Data are from the University College London Smoking Toolkit Study in England.

The prevalence of severe psychological distress was higher in 2020 compared with 2016/2017 among: those aged 65+, women, and among those with low cigarette addiction (Table 3). There were no apparent differences in the prevalence of psychological distress according to high cigarette addiction, recent quit attempts or motivation to stop smoking.

### 2.3 Discussion

Our first aim was to examine the prevalence of psychological distress among past-year smokers during April–July 2020 compared with April–July in 2016 and 2017. Our results indicate that between these time-periods in England there were increases in moderate and severe psychological distress, respectively, among both past-year and current smokers. Older age groups were less likely to report symptoms compared with younger groups, but there was no overall interaction between age and time period. The second aim of this study was to examine the distribution of psychological distress within sociodemographic and smoking characteristic categories of past-year smokers. Moderate psychological distress was greater in 2020 among those aged 16–24 and 45–54 years, women, those from more disadvantaged social grades, those with and without children at home and those with low cigarette addiction. Severe psychological distress was greater in 2020 among those aged 65+, women and among those with low cigarette addiction.

The increase in levels of both moderate and severe psychological distress among smokers is likely influenced by the ongoing COVID-19 pandemic and associated restrictions in England (Bu et al., 2020). Moreover, while mental health has deteriorated in the overall population as a result of COVID-19 (Pierce et al., 2020), our analysis using exclusively April–July 2020 data highlighted that smokers specifically continue to display elevated levels of psychological distress compared with non-smokers. Together these findings have concerning implications for existing smoking-related health inequalities considering the strong and potentially bi-directional associations between smoking and mental illness (Fluharty et al., 2017).

Older smokers were less likely to report psychological distress compared with younger groups (Brose et al., 2020). These findings re-emphasise the need to address higher prevalence of poor mental health among younger smokers (Pierce et al., 2020, Pedersen and Von Soest, 2009, Schmidt et al., 2019). We hypothesised that there may be an interaction between age-group and year with older smokers experiencing greater psychological distress in 2020 due to the age gradient in deaths from COVID-19 and the known risks of smoking. This was not borne out in the primary analysis, but there were signals of age-group differences in our stratified socio-demographic analyses discussed below.

Compared with 2016/17 the distribution of moderate psychological distress among smokers in 2020 was higher in those aged 16–24 and 45–54, women, more disadvantaged social grades and in those with or without children in the home. The distribution of severe psychological distress was broadly similar between the time periods, with the exceptions of higher prevalence in 2020 within women and those aged 65+. These demographic profiles of psychological distress have implications for existing inequalities and support findings that the impacts of COVID-19 on mental health have not been felt equally across society, but specifically among women and the more socioeconomically disadvantaged (Pierce et al., 2020). However, the reported significance of these stratified socio-demographic analyses within age (given the aforementioned absence of interaction effects), and other characteristics should be viewed descriptively.

Regarding smoking and quitting behaviour, between 2016/17 and 2020 there were no differences in the prevalence of high cigarette addiction among those with moderate or severe psychological distress, respectively. There were, however, increases in the prevalence of low addiction. Reasons for this are unclear but may reflect previous findings where smokers in general in England appear to have become less dependent on cigarettes in recent years (Garnett et al., 2020). Past month quit attempts or motivation to stop smoking was similar between 2016/17 and 2020 within any category of psychological distress. This is consistent with recent findings showing that COVID-19 triggered a minority of quit attempts in England (Heerfordt and Heerfordt, 2020, Tattan-Birch et al., 2020).

Recent YouGov data in England has suggested that those with existing mental health problems may have been more likely to have quit successfully during the pandemic (Action on Smoking and Health, 2020), but smokers with poor mental health who didn’t quit during the pandemic are smoking more and are less likely to quit as a result of COVID-19. As with the general population of smokers in England where quit attempts and short-term quit success have risen during 2020 (www.smokinginengland.info), further monitoring of whether these have translated into longer-term (i.e. >1 year) smoking abstinence is needed.

The levels of psychological distress among disadvantaged social grades and women, and the persistence of poor mental health among younger smokers is concerning because the prevalence of mental illness 2016/17 was already greater in these demographics than in previous years (Slee et al., xxxx). Mental health practitioners should continue to monitor the smoking status of their patients, and offer referral to local authority stop smoking services where they can receive effective support for smoking cessation (Gilbody et al., 2019). The majority of delivery of smoking cessation support has moved to telephone or online during the pandemic, and effective sources of digital support for smoking cessation should also be promoted (Taylor et al., 2017, Whittaker et al., 2019). Specific attention should be considered for smokers aged 65 + at this time, who are generally more dependent on cigarettes (Hall et al., 2008) and from our analyses appear to have greater psychological distress than previous years. Advice on effective harm reduction alternatives such as electronic cigarettes should also be considered (Hartmann_Boyce et al., 2020, Hickling et al., 2019) alongside clear public health messaging about the immediate health benefits of smoking cessation.

This study is limited by the use of cross-sectional survey data where smoking status is self-reported, and by the change in data collection from face-to-face to telephone in April 2020. Moreover, we did not have mental health data in 2018–2019 and the first three months of 2020. However, data from the opinions and lifestyle survey collected since 2018 highlight a deterioration in wellbeing among smokers following the onset of the pandemic in England (Public Health England, 2020). This study could not adjust for potential confounders (marital status, disability and education) which may have been associated with smoking and increases in distress during the pandemic.

Future longitudinal research should monitor changes in psychological distress among smokers throughout the pandemic and its aftermath. Greater understanding about the direction(s) of the relationship between smoking (including measures of smoking life-history) and mental illness, will inform the best approach for reducing smoking levels.

This exploratory study aimed to examine psychological distress in England among past-year smokers during April–July 2020 compared with the same time period in 2016 and 2017 and found there were increases in moderate and severe psychological distress.

## 3 Funding and acknowledgements

We are grateful to Cancer Research UK and the UK Prevention Research Partnership for funding the study. Authors are members of the UK Prevention Research Partnership, an initiative funded by UK Research and Innovation Councils, the Department of Health and Social Care (England), and the UK devolved administrations and leading health research charities.

## 4 Contributors

SC, JB and LK conceived of the study. All authors contributed to the study analysis plan. LK conducted the analysis and write up. HTB conducted the spline analysis. All authors contributed to the final manuscript. LK is the guarantor of this work and, as such, had full access to all the data and take responsibility for the integrity of the data and the accuracy of the data analysis.

## Declaration of Competing Interest

The authors declare the following financial interests/personal relationships which may be considered as potential competing interests: Authors are members of the UK Prevention Research Partnership, an initiative funded by UK Research and Innovation Councils, the Department of Health and Social Care (England), and the UK devolved administrations and leading health research charities. JB reports receiving grants from Cancer Research UK during the conduct of the study and receiving unrestricted research funding from pharmaceutical companies who manufacture smoking cessation medications to study smoking cessation outside the submitted work. LS reports receiving honoraria for talks, receiving an unrestricted research grant and travel expenses to attend meetings and workshops by pharmaceutical companies that make smoking cessation products (Pfizer and Johnson & Johnson), and acting as a paid reviewer for grant-awarding bodies and as a paid consultant for health care companies. LK, LB, GM and MH have no competing interests to declare.
