# Supplementary material for: Smoking, distress and COVID-19 in England: Cross-sectional population surveys from 2016 to 2020
Source: Prev Med Rep. 2021 May 29;23:101420. doi: 10.1016/j.pmedr.2021.101420 (PMC8193154; doi:10.1016/j.pmedr.2021.101420)
Supplement: Supplementary data 1 [file mmc1.docx]

**APPENDIX**

**Table A1**: Associations between i) moderate and ii) severe psychological distress and time period of survey (April-July 2020 vs all months in 2016-2017) among past-year smokers in England

|  | **Past-year smokers** | | | |
| --- | --- | --- | --- | --- |
|  | **Moderate distress^a^** | ***P*** | **Severe distress^b^** | ***P*** |
|  | (n=6,501) |  | (n=5,524) |  |
| **Time period** |  |  |  |  |
| 2016-2017 ref | 1 [Reference] |  | 1 [Reference] |  |
| 2020 | 2.02 (1.36-3.01) | **<.001** | 2.10 (1.17-3.64) | **.01** |
| **Age** |  |  |  |  |
| 16-25 | 1 [Reference] |  | 1 [Reference] |  |
| 25-34 | 0.69 (0.57-0.83) | **<.001** | 0.71 (0.54-0.95) | **.02** |
| 35-44 | 0.72 (0.59-0.88) | **.001** | 0.64 (0.47-0.86) | **.004** |
| 45-54 | 0.53 (0.43-0.65) | **<.001** | 0.58 (0.43-0.78) | **<.001** |
| 55-64 | 0.41 (0.33-0.52) | **<.001** | 0.40 (0.28-0.56) | **<.001** |
| 65+ | 0.26 (0.2-0.33) | **<.001** | 0.10 (0.06-0.16) | **<.001** |
| **Interaction terms** |  |  |  |  |
| 2020*25-34 | 0.77 (0.46-1.28) | .31 | 0.79 (0.38-1.66) | .53 |
| 2020*35-44 | 0.68 (0.38-1.22) | .20 | 0.86 (0.38-1.96) | .73 |
| 2020*45-54 | 0.85 (0.48-1.5) | .57 | 0.52 (0.2-1.26) | .15 |
| 2020*55-64 | 0.70 (0.38-1.26) | .24 | 0.53 (0.2-1.31) | .18 |
| 2020*65+ | 0.73 (0.37-1.40) | .35 | 1.6 (0.54-4.49) | .38 |

Ns are not weighted. All models are adjusted for age, sex and region. ^a^Sample includes past-year smokers with moderate (n=1,593) and none/minimal (n=5,003) distress; ^b^Sample includes past-year smokers with severe (n=599) and none/minimal (n=5,003) distress. Models are adjusted for age, sex and region.

**Post-hoc analyses**

**Table A2**: Associations between i) moderate and ii) severe psychological distress and time period of survey (April-July 2020 vs April-July 2016-2017) among recent ex-smokers in England

|  | **Moderate distress^a^** | ***P*** | **Severe distress^b^** | ***P*** |
| --- | --- | --- | --- | --- |
|  | (n=251) |  | (n=198) |  |
| **Time period** |  |  |  |  |
| 2016-2017 ref | 1 [Reference] |  | 1 [Reference] |  |
| 2020 | 1.33 (0.73-2.42) | .39 | 1.28 (0.48-3.41) | .62 |
|  |  |  |  |  |
| **Age** |  |  |  |  |
| 16-25 | 1 [Reference] |  | 1 [Reference] |  |
| 25-34 | 0.85 (0.37-1.92) | .69 | 0.55 (0.16-1.91) | .35 |
| 35-44 | 0.43 (0.16-1.09) | .08 | 0.09 (0-0.58) | **.03** |
| 45-54 | 0.41 (0.15-1.11) | .08 | 0.41 (0.09-1.64) | .22 |
| 55-64 | 0.30 (0.09-0.90) | **.04** | 0.65 (0.14-2.6) | .55 |
| 65+ | 0.15 (0.04-0.49) | **.003** | 0.24 (0.03-1.13) | .10 |

Model adjusted for social grade, sex, and region

**Table A3**: Bayes factor calculation for associations between moderate and severe psychological distress and time period of survey among recent ex-smokers in England.

| **Moderate Distress** |  |
| --- | --- |
| **Expected effect size OR = 1.1** |  |
| Sample standard error | 0.30 |
| Obtained sample estimate | 0.29 |
| Mean of alternative hypothesis (half-normal) | 0 |
| Plausible expected value | 0.095 |
| Number of tails | 1 |
| Bayes factor | 1.21 |
| **Expected effect size OR = 1.5** |  |
| Sample standard error | 0.30 |
| Obtained sample estimate | 0.29 |
| Mean of alternative hypothesis (half-normal) | 0 |
| Plausible expected value | 0.41 |
| Number of tails | 1 |
| Bayes factor | 1.23 |
| **Expected effect size OR = 1.9** |  |
| Sample standard error | 0.30 |
| Obtained sample estimate | 0.29 |
| Mean of alternative hypothesis (half-normal) | 0 |
| Plausible expected value | 0.64 |
| Number of tails | 1 |
| Bayes factor | 0.98 |
| **Severe distress** |  |
| **Expected effect size OR = 1.1** |  |
| Sample standard error | 0.30 |
| Obtained sample estimate | 0.26 |
| Mean of alternative hypothesis (half-normal) | 0 |
| Plausible expected value | 0.095 |
| Number of tails | 1 |
| Bayes factor | 1.17 |
| **Expected effect size OR = 1.5** |  |
| Sample standard error | 0.30 |
| Obtained sample estimate | 0.26 |
| Mean of alternative hypothesis (half-normal) | 0 |
| Plausible expected value | 0.41 |
| Number of tails | 1 |
| Bayes factor | 1.10 |
| **Expected effect size OR = 1.9** |  |
| Sample standard error | 0.30 |
| Obtained sample estimate | 0.26 |
| Mean of alternative hypothesis (half-normal) | 0 |
| Plausible expected value | 0.64 |
| Number of tails | 1 |
| Bayes factor | 0.86 |

**Figure A1:** Prevalence of psychological distress by smoking status in 2020


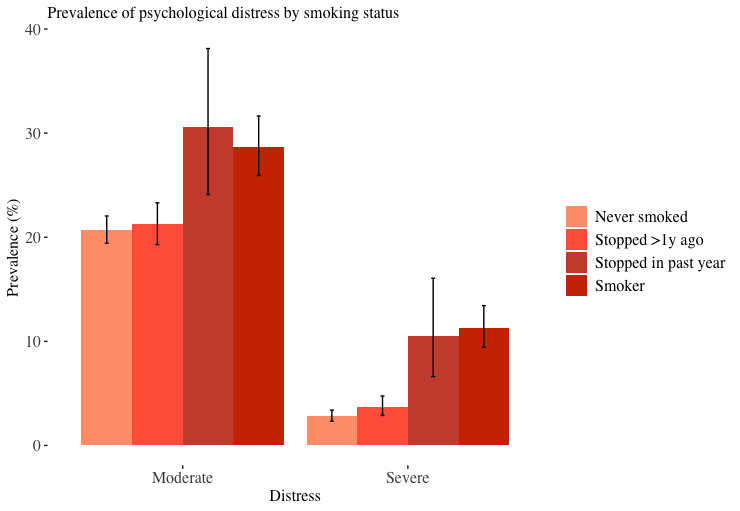


**Table A4**: Association between i) moderate and ii) severe psychological distress and smoking status between April-July 2020.

|  | **Moderate distress^a^** | ***P*** | **Severe distress^b^** | ***P*** |
| --- | --- | --- | --- | --- |
|  | (n=6192) |  | (n=5091) |  |
| **Smoking status** |  |  |  |  |
| Never smoked | 1 [Reference] |  | 1 [Reference] |  |
| Stopped >1y ago | 1.25 (1.07-1.46) | **.004** | 1.71 (1.19-2.43) | **.003** |
| Stopped past year | 1.61 (1.08-2.36) | **.02** | 4.84 (2.49-8.87) | **<.001** |
| Smoker | 1.46 (1.21-1.75) | **<.001** | 3.69 (2.64-5.15) | **<.001** |

Model is adjusted for age, sex, social grade and region.

**Psychological distress**

Participants were asked:

“During the past 30 days, about how often, if at all, did you feel... nervous; hopeless; restless or fidgety; so depressed that nothing could cheer you up; that everything was an effort; worthless?”

The answer options were presented in a randomised order and for each the respondent indicated one of the following: “All of the time (score=4); Most of the time (3); Some of the time (2); A little of the time (1); None of the time (0)”

A sum score with a possible range from 0-24 was calculated. The K6 has demonstrated utility to screen for severe psychological distress, but also for a moderate yet still clinically relevant level that warrants mental health intervention.22 Therefore, based on previous research scores of 13 and higher were categorised as severe psychological distress, scores between 5-12 as moderate and less than 5 as no/minimal psychological distress.22

**Motivation to stop smoking**

Motivation to stop smoking was assessed using the Motivation To Stop Scale, a single-item measure with seven response options representing increasing motivation to quit:

1. ‘I don’t want to stop smoking’

2. ‘I think I should stop smoking but don’t really want to’

3. ‘I want to stop smoking but haven’t thought about when’

4. ‘I REALLY want to stop smoking but I don’t know when I will’

5. ‘I want to stop smoking and hope to soon’

6. ‘I REALLY want to stop smoking and intend to in the next 3 months’

7. ‘I REALLY want to stop smoking and intend to in the next month’

Responses were collapsed into two variables reflecting high (6-7) vs. low or no motivation to stop smoking (1-5)

**Socio-demographic characteristics**

The socio-demographic variables age (categories 16-24, 25-34, 35-44, 45-54, 55-64, and ≥65 years), sex (categories women vs other), occupation-based social grade (AB (higher and intermediate managerial, administrative and professional), C1 (supervisory, clerical and junior managerial, administrative and professional), C2 (skilled manual workers), D (semi‐skilled and unskilled manual workers) and E (state pensioners, casual and lowest‐grade workers, unemployed)), region of England (government office region including nine categories: North East, North West, Yorkshire and the Humber, East Midlands, West Midlands, East of England, London, South East, South West), and the presence of children in the household were measured.

**Unregistered post-hoc analyses**

We conducted further logistic regression models to explore changes in moderate and severe psychological distress among recent ex-smokers (quit within the past year) between the two time periods (April-July 2020 vs April-July 2016-2017 as referent) and age (six categories with 16-24 as referent). We calculated Bayes factors (BF) for non-significant associations to explore whether they provided evidence for no effect (BF < 1/3) when compared to the alternative hypothesis or indicated data insensitivity (BF ≥ 1/3 and < 3). The alternative hypothesis was modelled using a half-normal distribution centred on zero, with a standard deviation equal to the expected effect size.

In 2016-2017 mental health data was only collected among current and recent ex-smokers. From April 2020 all respondents were asked questions about their mental health, allowing us to examine levels of psychological distress across all categories of smoking status (see Appendix A). Analysing psychological distress according to smoking status contextualises the findings among smokers against those who have never smoked or have been abstinent for a long time. Further analyses were run exploring differences in the prevalence psychological distress between April-July 2020 according to smoking status. The results are presented as weighted proportions (with 95% CIs). Logistic regression was used to estimate the association between psychological distress and smoking status (four categories: Smoker, Stopped in the past year, Stopped >1 year ago and Never Smoker (referent)).
